# Inbred or Outbred? Genetic Diversity in Laboratory Rodent Colonies

**DOI:** 10.1534/g3.117.300495

**Published:** 2017-12-13

**Authors:** Thomas D. Brekke, Katherine A. Steele, John F. Mulley

**Affiliations:** * School of Biological Sciences, Bangor University, LL57 2DG, United Kingdom; † School of Environment, Natural Resources and Geography, Bangor University, LL57 2DG, United Kingdom

**Keywords:** *Meriones*, genetic diversity, inbreeding, laboratory rodents, replication

## Abstract

Nonmodel rodents are widely used as subjects for both basic and applied biological research, but the genetic diversity of the study individuals is rarely quantified. University-housed colonies tend to be small and subject to founder effects and genetic drift; so they may be highly inbred or show substantial genetic divergence from other colonies, even those derived from the same source. Disregard for the levels of genetic diversity in an animal colony may result in a failure to replicate results if a different colony is used to repeat an experiment, as different colonies may have fixed alternative variants. Here we use high throughput sequencing to demonstrate genetic divergence in three isolated colonies of Mongolian gerbil (*Meriones unguiculatus*) even though they were all established recently from the same source. We also show that genetic diversity in allegedly “outbred” colonies of nonmodel rodents (gerbils, hamsters, house mice, deer mice, and rats) varies considerably from nearly no segregating diversity to very high levels of polymorphism. We conclude that genetic divergence in isolated colonies may play an important role in the “replication crisis.” In a more positive light, divergent rodent colonies represent an opportunity to leverage genetically distinct individuals in genetic crossing experiments. In sum, awareness of the genetic diversity of an animal colony is paramount as it allows researchers to properly replicate experiments and also to capitalize on other genetically distinct individuals to explore the genetic basis of a trait.

The genetic variation present in laboratory rodent colonies has important implications for the design, outcome, and reproducibility of biological experiments (Justice and Dhillon 2016). High levels of genetic variation reduce power and increase variation in the response to a treatment, but the experimental results may be more applicable to natural or human populations. Alternatively, inbred colonies provide more power and require fewer animals per experiment by limiting the noise caused by segregating genetic variation [here we define an inbred strain as the result of ≥20 generations of brother–sister mating or equivalent (Eppig 2007; Casellas 2011)]. Indeed, minimizing the number of animals [in accordance with the principle of *reduction* in the 3Rs (Russell and Burch 1959)] is one of the main reasons cited for the use of inbred lines rather than outbred colonies (Groen and Lagerwerf 1979; Festing 1999; Chia *et al.* 2005). Inbred lines with single genes knocked out have proven tremendously powerful for identifying the phenotypic effect of those genes (Festing 2010), but phenotypic traits and diseases often have complex genetic bases [*e.g.*, diabetes (Fuchsberger *et al.* 2016; Rich 2016) and epilepsy (Meisler *et al.* 2001)], so inbred models with no genetic variation may preclude a complete understanding of the underlying genetic architecture. Different epistatic interactions that occur in different genetic backgrounds may even reverse the phenotypic effect of a null allele (Sittig *et al.* 2016), making experimental design and the choice of inbred strain paramount (Little and Colegrave 2016). Genetic variation is essential for the identification of candidate genes underlying complex phenotypes, and projects such as the Collaborative Cross have gone to great effort (and expense) to capture variation from across multiple inbred mouse strains in a controlled manner (Churchill *et al.* 2004; Roberts *et al.* 2007; Collaborative Cross Consortium 2012; Threadgill and Churchill 2012). Such projects rely on the fact that although there is no segregating variation within a single inbred line, multiple inbred lines have fixed alternative variants and immense power can be gained by leveraging these fixed alleles in a genetic mapping experiment (Collaborative Cross Consortium 2012; Svenson *et al.* 2012; de Koning and McIntyre 2017).

Genetic crosses involving multiple inbred lines are hugely powerful for genetic experiments, but true inbred strains of mammals are rare outside of “model” rodents such as mice and rats. The use of “nonmodel” rodents, such as gerbils (*Meriones unguiculatus*; Stuermer and Wetzel 2006), hamsters (*Phodopus* sp.; Brekke *et al.* 2016), spiny mice (*Acomys* sp.; Gawriluk *et al.* 2016), and deer mice (*Peromyscus* sp.; Weber *et al.* 2013), is mainly restricted to outbred colonies with standing genetic variation. While significant steps are being taken to survey and characterize genetic diversity in outbred strains of house mice (Yalcin *et al.* 2010), surprisingly little work has been done to quantify diversity in colonies of nonmodel rodents. Indeed, the labeling of a strain of animals as “outbred” (Chia *et al.* 2005) or “wild derived” (Harper 2008), or even using a breeding scheme designed to minimize inbreeding (Yalcin *et al.* 2010), may have little to no bearing on the genetic diversity present. Instead, such labels only demonstrate that the animals have not *purposely* undergone the ≥20 generations of brother–sister mating necessary to purge segregating variation and establish a true inbred line (Eppig 2007; Casellas 2011). Whereas it is recognized that large colonies will slow the loss of genetic variation through drift (Papaioannou and Festing 1980), and commercial providers of outbred animals may maintain 50–100 or more breeding pairs per colony (Yalcin *et al.* 2010), the size of colonies especially in academic institutions is constrained by housing space, finances, and human resources. Furthermore, bottleneck or founder effects are likely to occur as animals are moved between colonies, used to establish a new colony, or rederive an old one. Thus, we should expect the amount of standing genetic variation to differ even between colonies of the same species and strain.

Mongolian gerbils (*M. unguiculatus*) are a common nonmodel rodent that have been used in biological research for many years and have informed our understanding of diseases such as epilepsy (Buchhalter 1993; Buckmaster 2006), stroke (Vincent and Rodrick 1979), and diabetes (Li *et al.* 2016); as well as basic biology questions about thermal regulation (Thiessen and Kittrell 1980; Wang *et al.* 2000), desert adaptation (McManus 1972), domestication (Stuermer *et al.* 2003; Stuermer and Wetzel 2006), reproductive biology (Clark *et al.* 1994), hearing (Chen *et al.* 2012; Abbas and Rivolta 2015), and more.

Despite the widespread use of gerbils in scientific research, few widely accessible transcriptomic and genomic resources have been developed, and the small numbers of genetic markers available are limited in their ability to accurately reveal genome-wide levels of diversity. Early reports using microsatellites (Neumann *et al.* 2001) suggested that genetic diversity in laboratory gerbil colonies is a small fraction of that in the wild and lower than the diversity captured across multiple inbred mouse (Love *et al.* 1990) or rat strains (Serikawa *et al.* 1992). Similarly, Razzoli (2003) found fairly low levels of heterozygosity using AFLPs. But more recent reports using microsatellites suggest that variation may be high, even similar to the levels found in the wild (Du *et al.* 2010, 2015). A simple explanation for this contradiction is that different strains of animals were used in each study. Du *et al.* (2010, 2015) surveyed four laboratory colonies from China, all of which were recently established from wild-caught individuals; whereas Neumann *et al.* (2001) and Razzoli (2003) mainly used animals originating from the Tumblebrook Farm strain. This strain has its origins in 20 pairs of wild-caught animals that were used to establish a colony in the Kitasato Institute in Japan in 1935. Since then, it has been rederived, sold, bottlenecked, and transferred repeatedly, beginning with a move to the Central Laboratories for Experimental Animals in 1949 (Petrij *et al.* 2001). From there, 11 animals were moved to the United States at Tumblebrook Farm in 1954 (Stuermer *et al.* 2003) and then again to Charles River Ltd. in Italy in 1996 (Neumann *et al.* 2001; Razzoli 2003), where the colony was rederived and has been maintained since with ≥100 breeding pairs and an outbred crossing scheme (C. Parady, personal communication). The population history of laboratory gerbils is thus punctuated by a series of bottleneck events each time the colony was moved and rederived. Thus, there seems to be a discrepancy in how these gerbils are maintained and sold by commercial providers (as a highly diverse outbred stock) and the results of previous genetic analyses [which suggest very low levels of diversity (Neumann *et al.* 2001; Razzoli 2003) and heterozygosity (Razzoli 2003)].

If laboratory gerbils are inbred, fewer are needed to achieve statistically significant results and maintain a breeding colony, but any results obtained may not be widely generalizable to other strains or species (Little and Colegrave 2016). Given the limitations of small-scale microsatellite and AFLP experiments, we therefore decided to use a genome-wide approach to quantify the genetic diversity present in Tumblebrook Farm-strain gerbils. Here, we evaluate patterns of standing genetic variation in animals from three different gerbil colonies to identify differences that may stem from a history of bottlenecks and isolation. All three colonies originated from the European colony managed by Charles River Ltd. We also compared these with the recently released whole genome sequence of an individual from an American stock of the Tumblebrook Farm strain (GenBank accession GCA_002204375.1). We interpret the levels of genetic variation in gerbils in comparison with colonies of other species such as house mice (*Mus musculus* subsp.), hamsters (*Phodopus* sp.), deer mice (*Peromyscus* sp.), and rats (*Rattus norvegicus*). We also discuss the possibility of leveraging the inescapable genetic drift in small mammal colonies to identify differentiated genetic markers for use in genetic mapping and association studies.

## Materials and Methods

### Animals

Mongolian gerbils are listed in Annex 1 of European Union (EU) Directive 2010/63/EU and must therefore be purposely bred for scientific research. The majority (if not all) gerbils used in the EU are derived from the Tumblebrook Farm stock and many academic institutions in the UK, and elsewhere, maintain their own colonies derived from these animals. We analyzed animals from three of these colonies, and to avoid confusion we refer to each colony by the name of the city in which it was first established: Edinburgh, Sheffield, and Bangor. The Edinburgh colony was established by Dr. Judith Allen at the University of Edinburgh *circa* 2005. In 2014, Dr. Leila Abbas established a new colony at the University of Sheffield from the Charles River Ltd. Tumblebrook stock and at the same time took over care and housing of three to four pairs of animals from Edinburgh, with both stocks maintained separately. In 2016, we took delivery of 12 new Tumblebrook animals from Charles River (seven female and five male) to establish the Bangor colony. We also received five animals from each of the Edinburgh (three female and two male) and Sheffield (two female and three male) stocks. All three groups were maintained in isolation in Bangor, except for a single testcross between an Edinburgh female and a Sheffield male. All animals were housed in accordance with EU and Home Office animal care regulations, and experiments were reviewed and approved by the Bangor University Animal Welfare and Ethical Review Board.

### Tissue collection, DNA extraction, library preparation, and sequencing

Liver tissue was collected from the 22 founder animals and two F_1_ Edinburgh × Sheffield offspring and was immediately snap frozen in liquid nitrogen after each animal was killed as part of routine colony management. DNA was extracted with the Qiagen DNeasy Blood and Tissue Kit and treated with RNase according to the manufacturer’s instructions. Extracted DNA was shipped to the Beijing Genome Institute (BGI) (Hong Kong) for library preparation and sequencing. Uniquely barcoded 100-bp, paired-end, genotyping-by-sequencing (GBS) libraries were prepared with the 5-bp cutter ApeKI, pooled, and sequenced on a single lane of Illumina 4000 (Elshire *et al.* 2011).

### Bioinformatics

BGI filtered the raw data through their SOAPnuke filter, which includes demultiplexing the reads, removing proprietary barcode sequences, and dropping reads that were >26% adapter sequence and/or where >40% of the bases were below a PHRED quality score of 15. We used the Stacks pipeline (v1.46; Catchen *et al.* 2011, 2013) to identify tags and call SNPs from the first reads, resulting in an average sequencing effort of 7.8 million reads per individual. This analysis included the standard Stacks pipeline components: process_radtags, ustacks, cstacks, sstacks, and populations. All scripts were run with default flags with the following exceptions: With process_radtags we cleaned reads (-c), discarded reads with low quality scores (-q), rescued radtags (-r), and truncated read length to 92 bases (-t 92) to avoid variation in read length that would otherwise disrupt the remaining pipeline. Thus, our final markers were all 92 bp long. We ran the deleveraging algorithm in ustacks (-d) and used six individuals (a female and male from each Bangor, Edinburgh, and Sheffield strains) for cstacks. We generated a reference fasta (Supplemental Material, File S1) from the output of cstacks and to it we aligned the raw reads with bwa mem (Li and Durbin 2009). From these alignments, we extracted depth of coverage with samtools (Li *et al.* 2009) to annotate autosomal, X-, and Y-linked markers. Coverage was standardized by the sequencing effort of each individual and multiplied by 1,000,000 before being summed across males and females. Sex linkage is apparent by comparing standardized coverage of each marker in males *vs.* females. We first annotated 422,664 markers with <10× total standardized coverage as “unknown” and removed these from the data set as their coverage is too low to reliably differentiate X- and Y-linked tags from autosomal tags or call variants. From the remaining 725,888 markers, we identified Y-linked ones as those with <1× standardized coverage in females. X-linked markers fulfilled the inequality: coverage^male^ < 3/4 coverage^female^ − 5. The slope of this line was chosen to discriminate points in the X-linked cluster (slope = 1/2) from those in the autosomal cluster (slope = 1). The intercept was chosen to remain fairly conservative near the origin; that is erring toward labeling a true X-linked tag as an autosome rather than labeling a true autosomal tag as X-linked. All remaining tags were annotated as autosomal. The populations script was used to generate diversity metrics and F statistics across the genome for autosomal, X-, and Y-linked markers. Genotypes were called only for individuals with >10× coverage (-m 10) and only for SNPs in the first 90 bases. We blacklisted SNPs in the final two bases because of an unusually high number of SNPs on those bases (for further discussion see Figure S1). Finally, we evaluated genetic similarity and population structure with the SNPRelate package in R (Zheng *et al.* 2012) and the program Structure (Pritchard *et al.* 2000; Rosenberg *et al.* 2002; Falush *et al.* 2003, 2007). We visualized structure data with the program distruct (Rosenberg 2003). We calculated pairwise diversity between our reference and the recently released gerbil whole genome sequence (GenBank accession GCA_002204375.1) by aligning the reference sequences to the genome with bwa mem, discarding partial-length alignments, and counting mismatches across the first 90 bases of the reference. Finally, we determined how many SNP-containing GBS tags were found in coding regions by comparing the alignment locations of the tags with the gene annotations in the gerbil genome.

To evaluate the levels of nucleotide diversity in gerbils in a more general sense, we downloaded restriction-site associated DNA (RAD) sequencing data from deer mice (SRA accession PRJNA186607; Weber *et al.* 2013) and were provided with RAD sequence data for hamsters (J. Good, personal communication). These RAD data sets were analyzed with the Stacks pipeline described above (omitting the chromosomal annotation steps) to be directly comparable with diversity estimates in gerbils. To provide further context for our estimates of nucleotide diversity (π), we also retrieved recently published diversity metrics from house mice in the Collaborative Cross (Srivastava *et al.* 2017), various inbred and wild-caught mouse colonies (Salcedo *et al.* 2007), and inbred and wild-caught rats (Smits *et al.* 2004; Ness *et al.* 2012).

### Data availability

Raw sequencing data are archived in the SRA under the BioProject accession number PRJNA397533 and the sample accession numbers SAMN07460176–SAMN07460199. The reference fasta file (including autosomal, X-, or Y-linkage of each tag) as well as the VCF file containing the locations of all SNPs are available as File S1.

## Results

**Figure 1:**
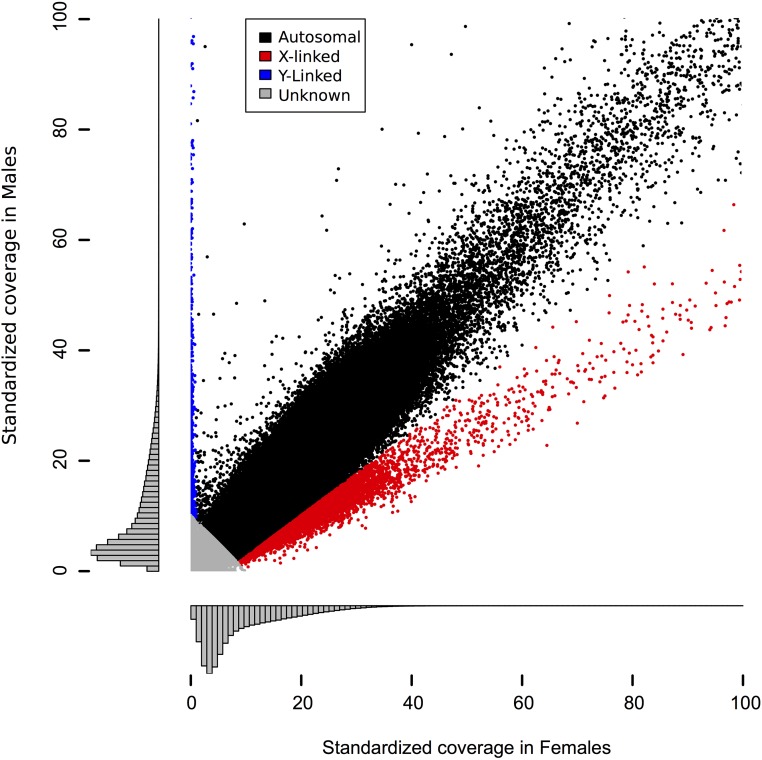
Relative coverage in females and males can be used to identify sex chromosomes. Plotted here is standardized coverage in females against standardized coverage in males; histograms show the density of markers along each axis. The markers shown are those with low overall coverage; a long tail exists in both females and males and is not shown here. Markers with <10× total standardized coverage were annotated as unknown (gray) because those have too little coverage to reliably distinguish X- and Y-linkage from autosomes. Of the remaining tags, those with <1× standardized coverage in females are annotated as Y-linked (blue). Those that satisfy the inequality: coverage^male^ < 3/4 coverage^female^ − 5 were identified as X-linked (red). This line has a slope designed to discriminate points in the X-linked cluster (slope = 1/2) from those in the autosomal cluster (slope = 1) while remaining fairly conservative near the origin. All remaining tags were annotated as autosomal (black).

We compared sequencing coverage in females and males to annotate 718,385 autosomal markers, 5148 X-linked markers, and 2355 Y-linked markers (Figure 1). We identified 30,365 SNPs spread across 24,326 markers (1.25 SNPs per marker). Average π is 0.0059 (Table 1). Most SNP-containing markers (∼95%) are intergenic and so this value tends to describe variation in unconstrained, noncoding regions. Around 5% of SNP-containing markers (1186) are found in 1109 different genes and these are listed in Table S1. Average heterozygosity at autosomal variant sites is 0.447 and is slightly higher on the sex chromosomes (Table 1).

**Table 1:** Diversity metrics in gerbils across the genome

Colony	*N*	Autosomal					X-Linked					Y-Linked				
		Bases	Private	Poly.	π	Het.	Bases	Private	Poly.	π	Het.	Bases	Private	Poly.	π	Het.
Bangor	12	2,131,103	5702	24,516	0.0054	0.459	53,540	134	822	0.0039	0.619	32,014	32	489	0.0067	0.809
Sheffield	5	1,485,832	592	11,409	0.0049	0.421	45,414	26	547	0.0060	0.630	20,850	12	265	0.0086	0.772
Edinburgh	5	1,688,545	1336	8,422	0.0032	0.313	50,487	40	564	0.0046	0.558	18,560	20	253	0.0100	0.846
All colonies and F_1_’s	24	2,150,776	n/a	28,885	0.0059	0.447	54,464	n/a	929	0.0036	0.606	32,752	n/a	551	0.0093	0.806

*N*, number of individuals evaluated; bases, number of bases with sufficient coverage to evaluate nucleotide diversity; private, number of sites with private alleles; poly., number of sites that are polymorphic; π, nucleotide diversity in each colony; het., average heterozygosity at the polymorphic sites.

To evaluate how different the gerbil genome (GCA_002204375.1) is from our colonies, we counted the number of differences between our GBS reference and the genome. Full-length alignments were found for 674,342 (93%) of our reference sequences when aligned to the genome. We found 47,223 single-base differences in these aligned regions, far more than the SNPs that segregate within the colonies we assayed. This pattern is consistent with the known population history of laboratory gerbils: the Charles River colony, from which our animals originate, was rederived from a United States colony from which the DNA for the genome was supplied.

**Figure 2:**
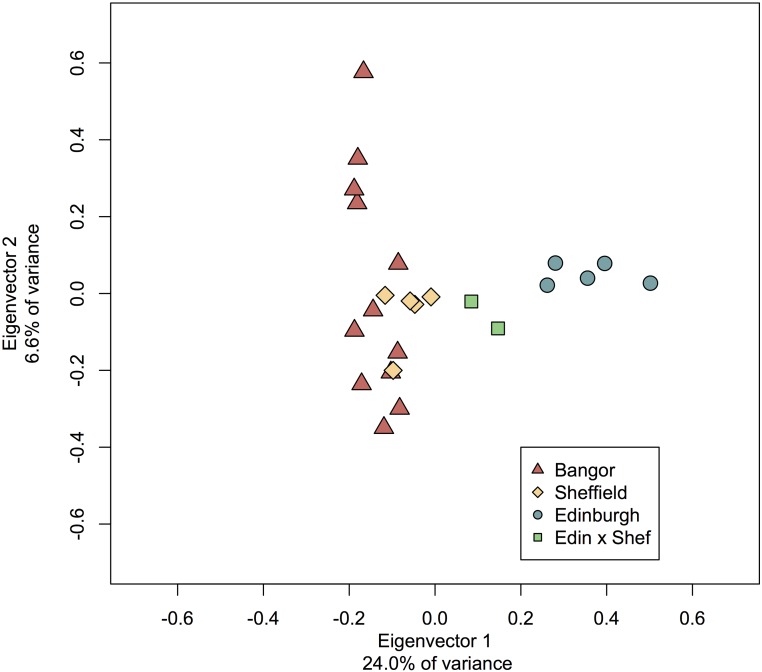
PCA of 28,885 autosomal SNPs. Eigenvector 1 explains the majority of the diversity in these samples and strongly differentiates the Edinburgh colony from the others. Sheffield contains a subset of the genetic diversity found within Bangor colony. F_1_ offspring between Edinburgh female and Sheffield male are intermediate.

**Figure 3:**
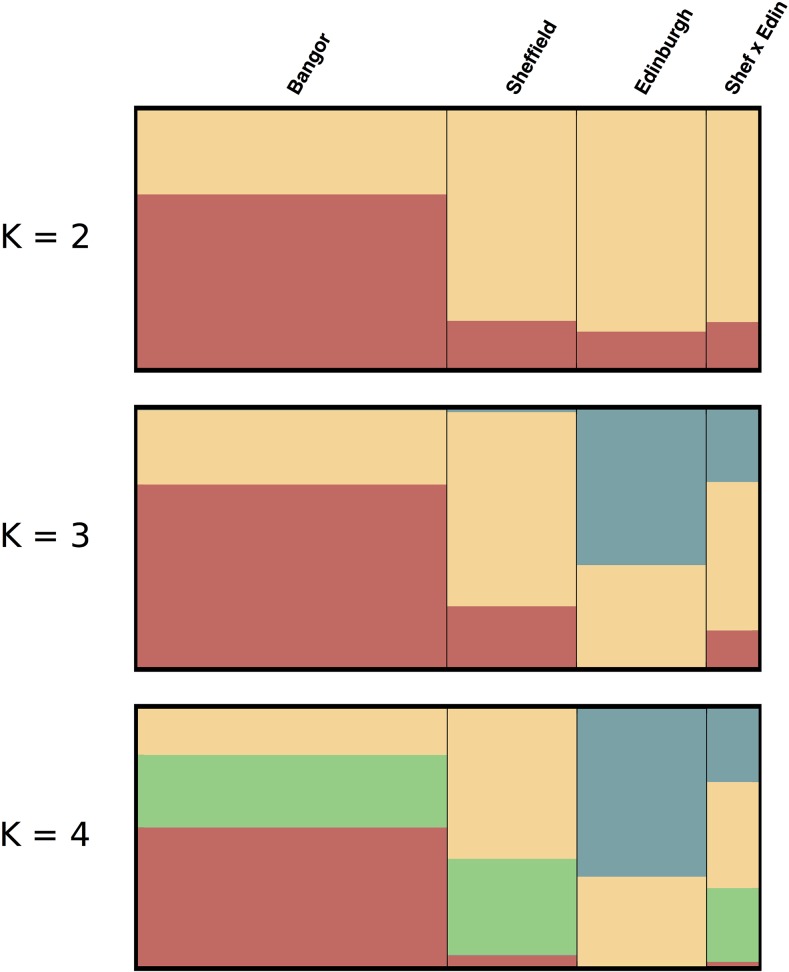
Structure plots of gerbil colonies. Structure was run for 20,000 iterations with 10,000 iterations of burn-in for *K* = 2, 3, and 4. In general, the different colonies have similar assignment especially at low *K* values. Edinburgh animals fall out uniquely at higher *K* values (*i.e.*, blue). The F_1_ hybrids have mixed ancestry as expected for the offspring of a Sheffield × Edinburgh cross.

We used 28,885 autosomal SNPs to evaluate the diversity between the three colonies and found that whereas each colony does possess a small set of private alleles, most alleles are shared across all colonies (Table 1). A substantial portion of the variation (24%) is explained by differences between Edinburgh and the other colonies (Figure 2). Eigenvector 2 shows that much of the remaining variation (7%) segregates within the Bangor colony. No higher-order eigenvectors discriminate the colonies, instead they partition variation that is common to all. F_st_ metrics between the colonies suggest a high overall similarity between Bangor and Sheffield (F_st_ = 0.069) and identify Edinburgh as an outlier with an F_st_ of 0.235 when compared with Bangor and 0.352 when compared with Sheffield. The structure analysis also suggests little overall differentiation between Bangor and Sheffield, and finds that Edinburgh is slightly more divergent, though still very similar (Figure 3). Overall, these data suggest that although Edinburgh animals have marked differences from other gerbils, they still share many genetic variants.

In general, the highest diversity is found in the Bangor animals. This is apparent in both the principal component analysis (PCA) (Figure 2) and the number of polymorphic sites segregating within the Bangor strain (Table 1) and may be due to the higher number of individuals screened or to the fact that they have not been maintained at low population sizes like the animals from Sheffield and Edinburgh. Edinburgh animals have far fewer segregating sites, lower nucleotide diversity, and lower heterozygosity overall, which may be a result of serial bottlenecks. The PCA and the low number of private alleles suggest that the Sheffield animals contain a subset of the diversity found within the Bangor strain (Figure 2). As expected, the F_1_ offspring between Edinburgh and Sheffield are found to be intermediate to the parents.

Although genetic diversity in laboratory gerbils (π = 0.0059) is lower than that captured in the mice of the Collaborative Cross (π = 0.0289; Srivastava *et al.* 2017), gerbil diversity is quite high compared with many other laboratory rodents (Table 2). In fact, gerbils rival the diversity found in wild-derived mouse colonies [*M. musculus*: π = 0.0054; *M. domesticus*: π = 0.0102 (Salcedo *et al.* 2007)] and, contrary to previous claims (Neumann *et al.* 2001; Razzoli 2003), exceed the diversity in rats, whether wild caught (π = 0.0022; Ness *et al.* 2012) or between inbred strains (0.0029 > π > 0.0015; Smits *et al.* 2004). It is clear that genetic diversity in Tumblebrook gerbils is much higher than previous reports suggest (Neumann *et al.* 2001; Razzoli 2003). It is also clear that breeding scheme alone does not robustly predict the amount of standing genetic diversity of an animal colony (Yalcin *et al.* 2010).

**Table 2:** Nucleotide diversity in various rodent colonies

Species	π	Number of Individuals	Breeding Scheme	Region of Genome for Which π Was Evaluated	Approximate Number of Bases Surveyed	Citation
*M. unguiculatus*	0.0059	24	Outbred colony	Near autosomal restriction sites (GBS)	2,200,000	This article
*M. musculus* subsp.*^a^*	0.0289	69	Collaborative Cross	Whole genome sequencing	2,300,000,000	Srivastava *et al.* (2017)
*M. musculus* subsp.	0.0055	8	Eight inbred strains	Autosomal genes (Sanger)	14,000	Salcedo *et al.* (2007)
*M. musculus domesticus*	0.0102	64	Wild caught	Autosomal genes (Sanger)	15,000	Salcedo *et al.* (2007)
*M. musculus musculus*	0.0054	26	Wild caught	Autosomal genes (Sanger)	15,000	Salcedo *et al.* (2007)
*Peromyscus maniculatus**^b^*	0.0006	13	Outbred colony	Near restriction sites (ddRAD)	400,000	Weber *et al.* (2013)
*P. polionotus**^b^*	0.0010	1	Outbred colony	Near restriction sites (ddRAD)	400,000	Weber *et al.* (2013)
*Phodopus campbelli**^c^*	0.0006	14	Outbred colony	Near restriction sites (ddRAD)	1,300,000	J. Good, personal communication
*P. sungorus**^c^*	0.0002	11	Outbred colony	Near restriction sites (ddRAD)	1,400,000	J. Good, personal communication
*R. norvegicus*	0.0015–0.0029	96	96 inbred strains	CEL I-based SNP detection	5,800,000	Smits *et al.* (2004)
*R. norvegicus*	0.0022	58	Wild caught	Autosomal genes (Sanger)	10,000	Ness *et al.* (2012)

a Based on mean value of 69 Collaborative Cross individuals. Data from the column titled “% het (autosomes) in sequenced sample” in table S2 of Srivastava *et al.* (2017). Approximate number of bases surveyed is based on a genome size of 2.7 GB divided by the mean “% coverage at 15×.”

b The *Peromyscus* animals evaluated here are the BW and PO strains originating with the *Peromyscus* stock center, bred at Harvard University in the Hoekstra laboratory, and sequenced by Weber *et al.* (2013).

c The *Phodopus* animals evaluated here are from the Good laboratory at the University of Montana described in Brekke and Good (2014).

## Discussion

We have demonstrated that appreciable genetic diversity segregates within Tumblebrook Farm-strain Mongolian gerbils. Our findings are contrary to earlier reports suggesting that diversity may be as low as, or lower than, laboratory mouse colonies (Neumann *et al.* 2001; Razzoli 2003). These reports evaluated a small portion of the genome and, perhaps unsurprisingly, found little variation. For instance, Neumann *et al.* (2001) evaluated diversity at nine microsatellites and found that laboratory strains had severely reduced allelic diversity compared to wild animals, and Razzoli (2003) found low heterozygosity using 228 AFLP fragments from six primer combinations. Our genome-wide assay evaluated millions of bases and so has much higher power to find rare variants. Using these data, we find that genetic diversity in Mongolian gerbils is relatively high among outbred nonmodel rodent colonies (π = 0.0059 in gerbils and π ≤ 0.0010 in other nonmodel rodents, Table 2).

Laboratory-maintained rodent colonies are often small due to the costs and space needed for maintenance of many animals. With such small populations, genetic drift plays an important role in determining the standing level of variation. Drift can be expected to increase genetic differentiation between colonies through time, especially given the population bottleneck that often occurs when a colony is established or moved to a new location. Knowledge of levels of genetic diversity in an institutional colony is therefore vital for correct colony management; for example, *Phodopus* hamsters have been referred to as outbred and maintained in large colonies (Brekke and Good 2014). However, analysis of double-digest RAD data from two hamster species (J. Good, personal communication) shows that, in fact, genetic diversity is extremely low in both (Table 2), and so hamster colonies could be maintained with few individuals with no resulting loss of diversity. Despite the length of time in captivity, the Tumblebrook gerbils are (correctly) maintained as a large outbred colony (≥100 breeding pairs) by Charles River Ltd. Our data suggest that the diversity present in that original stock has been subsampled and exposed to drift in each of the three independent colonies we assayed. At one extreme is the Edinburgh colony which was not only the first to be isolated from Tumblebrook, but has been transferred through three universities and experienced the associated bottlenecks. Given this history, it is not surprising that the Edinburgh animals have the fewest SNPs segregating within them, nor that they are somewhat differentiated from Bangor and Sheffield. The Sheffield animals, which were established from Tumblebrook strain founders in 2014 and have been moved through only two universities, also show a reduced diversity, though still higher than Edinburgh. The Bangor colony was established most recently in 2016 and has the highest amount of diversity. As these animals were sent directly from Charles River Ltd., they likely represent a large portion of the variation contained in the Tumblebrook stock. Our data suggest that genetic drift in these three colonies is actively eroding the standing genetic variation and, as they have been maintained in isolation from each other, it has resulted in noticeable differentiation between the colonies.

There are two major ramifications of the loss and partitioning of genetic variation in laboratory colonies. First, animals from the same original outbred stock may respond very differently to an experiment if they come from different isolated colonies. Many articles state that diversity in gerbils is quite low, one even suggesting that smaller error bars in laboratory individuals than wild-caught individuals are due to the lower genetic diversity (*i.e.*, Stuermer and Wetzel 2006). Although it is almost certainly correct that the diversity in their colony is low, our data suggest that this is likely a reflection of high drift in an isolated colony rather than low diversity in the original Tumblebrook stocks or even across all laboratory gerbils in general. Low diversity is an important factor in interpreting many experimental results, but generally missing from this acknowledgment is that although diversity is likely low in any specific colony, that does not mean that all colonies are genetically similar. This may partly explain why some experimental outcomes are not able to be replicated despite using animals from the same original outbred strain (Richter *et al.* 2011; Justice and Dhillon 2016). This general argument is applicable not only to rodent colonies, but any laboratory animals of any taxa where the population size is limited.

The second important ramification of high genetic drift in laboratory colonies is that although diversity will be lost in any single colony through time, across multiple isolated colonies much of the original diversity may be preserved. This is not a new idea and there are major ongoing efforts using multiple inbred strains to capture the range of natural diversity (Churchill *et al.* 2004; Collaborative Cross Consortium 2012; Threadgill and Churchill 2012). By intercrossing between multiple differentiated colonies, researchers can do controlled experiments designed to uncover the genetic architecture of complex traits (Festing 2010). In this regard, gerbils seem to be good candidates. Indeed, the high overall variation, number of private alleles within colonies, and intermediate location of the Sheffield × Edinburgh F_1_ offspring in the PCA (Figure 2) all suggest that sufficient diversity exists between the colonies for successful genetic experiments.

### Conclusions

Despite being derived from a relatively small number of founders and experiencing repeated bottlenecks over the past 80 years in captivity, the Tumblebrook Farm strain of Mongolian gerbils does not have low levels of genetic diversity. However, we have shown that genetic drift in small institutional colonies has increased differentiation and such drift may affect the reproducibility of experiments. For those doing animal research, the choice of whether to use inbred or outbred animals depends on the specific question being addressed (Little and Colegrave 2016). For instance, a few strains of highly inbred animals may be best for identifying the function of a specific gene, while a highly outbred population that captures much of the natural variation may be better for understanding things like the population level response to climate change. Regardless of the question, it is important to know the genetic diversity of the animals. Thus, we advise that experimenters consider the history of their colony when planning and performing research projects using gerbils and other animal models. Specifically, as the label of “outbred” is an unreliable metric of genetic variation, we suggest that researchers using such colonies verify the diversity of their animals directly through sequencing. Suggestions have previously been made for the ideal number of markers to calculate relatedness (>125 but <500; Santure *et al.* 2010) and heterozygosity (>200; Balloux *et al.* 2004). However, with current sequencing prices, it is generally less expensive to use a GBS or RAD approach and assay tens of thousands of markers than to develop a microsatellite or Sanger panel for hundreds of markers. Either way, with a bit of sequencing, researchers will be able to quickly and reliably assay genetic diversity in their colonies and then make appropriate choices about which animals to use for which experiments. We further suggest that published claims on levels of genetic diversity in laboratory rodents based on small numbers of genetic markers should be taken with a pinch of salt.

## Supplementary Material

Supplemental material is available online at www.g3journal.org/lookup/suppl/doi:10.1534/g3.117.300495/-/DC1.
