# Supplementary material for: Inbred or Outbred? Genetic Diversity in Laboratory Rodent Colonies
Source: G3 (Bethesda). 2017 Dec 13;8(2):679–86. doi: 10.1534/g3.117.300495 (PMC5919727; doi:10.1534/g3.117.300495)
Supplement: Supplementary file 1 [file 679FigureS1.pdf]

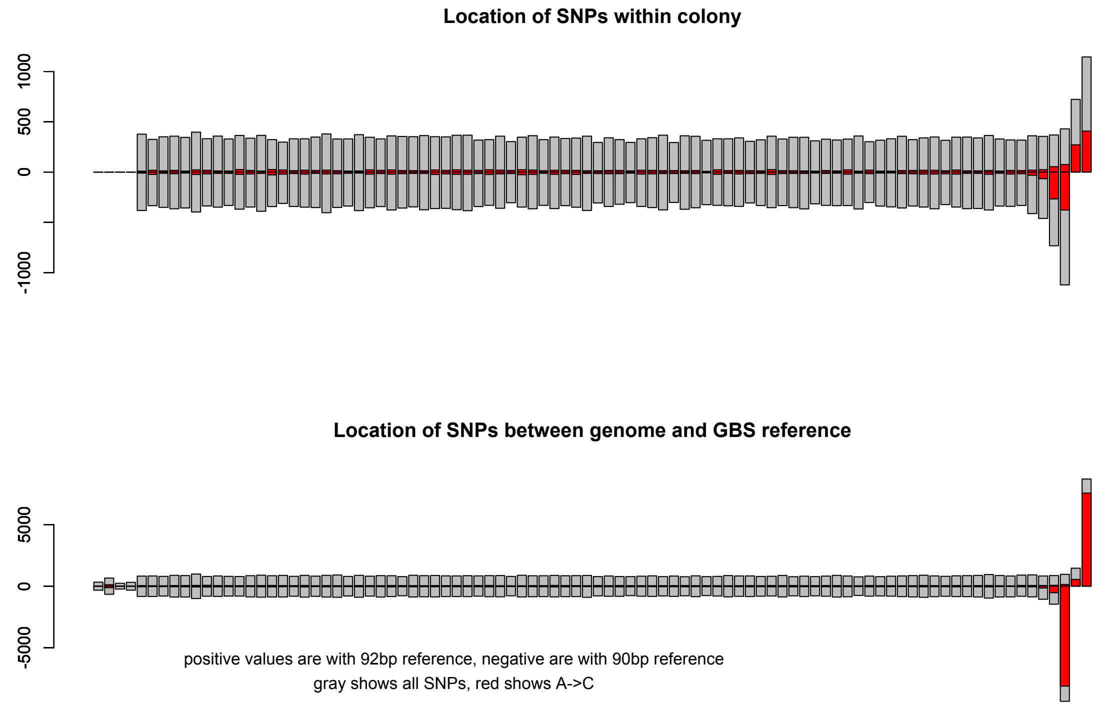

Figure S1. SNP Profiles

All of the entries in the reference we extracted from the STACKs pipeline are 92 bases long. This histogram shows the number of SNPs per base position within the animals we sequenced (top panel) and when compared with the Lucigen genome (bottom panel). The final two bases show unusually high numbers of SNPs in both cases, and especially high numbers of A to C transversions (red).

We found that these excess SNPs are always associated with a restriction cutsite. And specifically, they are associated with regions where the cutsite is within two bases from the end of the reference. That is, all the spurious cases ended with either CGWCX or CGWCXX where ‘X’ represents the SNP and CGWC is the restriction recognition sequence. Given the way in which GBS libraries are made, we believe that these SNPs may be part of the barcode and/or Illumina adapter sequence that was too short to be recognized and trimmed by BGI.

Our first attempt to deal with this issue was by cropping the reads entering the STACKs pipeline to 90 bases as we expected that would remove the short bits of adapter that remained. We tested this by re-running the STACKs pipeline using the new 90bp reference. Unfortunately, the issue was not resolved (see the negative values in both panels above). We found that the easiest way to remove these tailing SNPs was to use the 92bp reference, but blacklist the final two bases from the analyses of diversity (this was implemented in the populations script through a whitelist of SNPs in the first 90 bases rather than a blacklist of SNPs in the final 2 bases because populations does not allow SNP-specific blacklists).

Despite our solution, the question remained: Why do we find elevated substitution rates near the ends of the reads regardless of the length of the read? We think the answer lies in the fact that alignments only allow so many mismatches. For example, there are reads that have a cutsite four bases from the end of the read (CGWCXXXX), but four mismatched bases is too many to form an alignment (the tailing bases would be soft-clipped rather than aligned as a mismatch) and so these were never analyzed for SNPs (we counted SNPs only in alignments without any clipping). When we later trimmed the tailing two bases off, these cases now had only two mismatches in the alignment, and so passed the alignment-length filter, and the mismatches were identified as SNPs. As cutsites do occur randomly in the genome, it does not matter if we crop two or ten bases from the raw reads, a cutsite would still lie within two bases on some reference sequence. The best way perhaps to solve the issue would be to do a thorough bout of adapter trimming followed by cropping five to six bases. Unfortunately, we had the libraries built and sequenced by BGI who used proprietary barcodes and adapter sequences. As they were unwilling to share these sequences with us, further cleaning of the reads was not possible and the blacklisting approach that we used seems best.
